# HDAC and Ku70 axis- an effective target for apoptosis induction by a new 2-cyano-3-oxo-1,9-dien glycyrrhetinic acid analogue

**DOI:** 10.1038/s41419-018-0602-1

**Published:** 2018-05-24

**Authors:** Ping Gong, Kun Li, Ying Li, Dan Liu, Linxiang Zhao, Yongkui Jing

**Affiliations:** 1 Department of Pharmacology, Shenyang Pharmaceutical University, Shenyang, 110016 PR China; 2 Department of Medicinal Chemistry, Shenyang Pharmaceutical University, Shenyang, 110016 PR China; 3 Key Laboratory of Structure-Based Drug Design & Discovery of Ministry of Education, Shenyang Pharmaceutical University, Shenyang, 110016 PR China

## Abstract

Methyl 2-cyano-3,12-dioxo-18β-olean-1,9(11)-dien-30-oate (CDODO-Me, 10d) derived from glycyrrhetinic acid and methyl-2-cyano-3,12-dioxooleana-1,9-dien-28-oic acid (CDDO-Me) derived from oleanoic acid are potent apoptosis inducers developed to clinical trials. Both compounds have high affinity for reduced  glutathione (GSH), which needs to be overcome to improve their target selectivity. We generated a new 10d analogue methyl 2-cyano-3-oxo-18β-olean-1,9(11), 12-trien-30-oate (COOTO, 10e), which retains high apoptosis inducing ability, while displaying decreased affinity for GSH, and explored the acting targets. We found that it induces Noxa level, reduces c-Flip level and causes Bax/Bak activation. Silencing of either Noxa or Bak significantly attenuated apoptosis induction of 10e. We linked these events due to targeting HDAC3/HDAC6 and Ku70 axis. 10e treatment reduced the levels of HDAC3 and HDAC6 with increased DNA damage/repair marker gamma-H2AX (γ-H2AX) and acetylated Ku70. c-Flip dissociates from acetylated Ku70 undergoing degradation, while Bax dissociates from acetylated Ku70 undergoing activation. Silencing of either HDAC3 or HDAC6 enhanced 10e-induced apoptosis. We reveal a new action cascade of this category of compounds that involves targeting of HADC3/6 proteins and Ku70 acetylation.

## Introduction

**Fig. 1:**
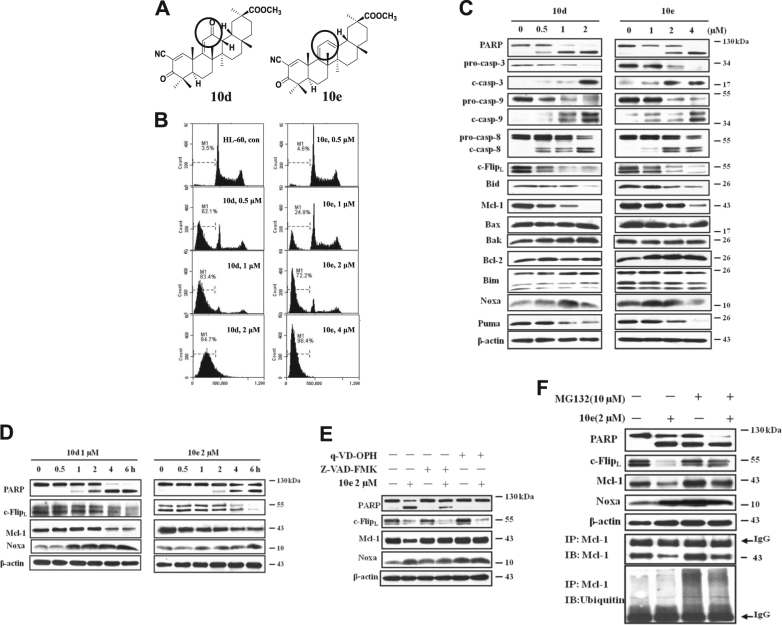
10d and 10e induce apoptosis in HL-60 cells associated with upregulation of Noxa and downregulation of Mcl-1 and c-Flip. **A** The chemical structures of 10d and 10e. **B** HL-60 cells were treated with 10d and 10e at the indicated concentrations for 6 h and the cells in subG1 phase were determined using FACS analysis after staining with PI. con, untreated cells. **C** HL-60 cells treated with 10d or 10e at different concentrations for 6 h, the relative levels of each protein were analyzed with western blotting using specific antibodies. **D** The HL-60 cells were treated with 1 μM 10d or 2 μM 10e for the indicated time, the levels of PARP, c-Flip_L_, Noxa proteins were examined. **E** HL-60 cells were pretreated with 100 μM Z-VAD-FMK or 25 μM q-VD-OPH for 1 h, following by treatment with 2 μM 10e for 6 h, the relative levels of each protein were analyzed with western blotting using specific antibodies. **F** HL-60 cells were pretreated with 10 μM MG132 for 1 h and then treated with or without 2 μM 10e for 4 h. The relative levels of PARP, c-Flip, Mcl-1, and Noxa were determined using Western blot analysis. The cell lysates was also immunoprecipitated with an anti-Mcl-1 antibody, and then immunoblotted with an anti-Mcl-1 antibody and an anti-ubiquitin antibody

18β-glycyrrhetinic acid (GA) is a naturally occurring oleanane-type pentacyclic triterpenoid isolated from the plant (*Glycyrrhiza glabra*) with antitumor activities^1^. We and other groups have performed structural modifications of this compound and obtained a derivative (methyl-2-cyano-3,12-dioxo-18β-olean-1,9(11)-dien-30-oate) (CDODO-Me) (Fig. 1a), with greater potency of apoptosis induction and tumor cell growth inhibition^2–5^. A similar compound, methyl-2-cyano-3,12-dioxooleana-1,9-dien-28-oic acid (CDDO-Me) was also derived from oleanoic acid and is currently being clinically tested as an anticancer agent^6,7^. The mechanism by which both CDODO-Me and CDDO-Me inhibit tumor growth and induce apoptosis^4,6,8^ has not been elucidated. Both compounds bind strongly to reduced glutathione (GSH) and deplete intracellular GSH^4,9–12^, which has been originally assumed to be one mechanism of apoptosis induction. GSH protects the cells from oxidative stress^13^. High concentrations of GSH, which is present in tumor cells and in human tissues might, by binding and neutralizing the therapeutic compounds, reduce their effectiveness hence the need to modify these compounds to reduce their binding to GSH^13^. To accomplish this goal, we further modified CDODO-Me at C ring. By converting the carbonyl group in C ring into a dioen structure and generated methyl-2-cyano-3-oxo-18β-olean-1,9(11), 12-trien-30-oate (COOTO-Me, 10e, Fig. 1A)^2^. This compound only has minor decreased ability of inducing apoptosis comparing to the compound 10d but its ability to deplete the intracellular GSH is significantly decreased^2^, providing an opportunity to identify the targets and the mechanisms of apoptosis induction without the involvement of GSH.

The published mechanisms of apoptosis induction by CDODO-Me and CDDO-Me include an increase in reactive oxygen species (ROS), and the downregulation of the anti-apoptotic proteins Mcl-1 and c-Flip^4,9,11^. We did not find a role for the increased ROS in apoptosis induction in cells treated with 10e; in spite of this, treatment with 10e decreased c-Flip and Mcl-1 levels. c-Flip is a key protein blocking caspase-8 activation, which controls extrinsic apoptosis pathway^14^, while Mcl-1 is one of the anti-apoptotic proteins controlling mitochondrial apoptotic pathway^15^. The latter is controlled by multiple anti- and pro-apoptotic proteins, and BH3-only proteins, which are currently being explored as therapeutic targets^16^. We propose that apoptosis induction by 10e and 10d, as well as CDDO-Me is mediated by a new mechanism that leads to destruction of several anti-apoptotic proteins. We explored this possibility by testing the key factors regulating both intrinsic and extrinsic apoptotic pathways in several human myeloid leukemia cell lines treated with 10e. Consistent with the reported downregulation of Mcl-1 and c-Flip, we found that Noxa was induced and that both Bax and Bak were activated. We further examined the mechanisms that might be responsible for the observed changes and found that the acetylation of Ku70 plays an essential role. We also found that 10e induces Ku70 acetylation by down-regulating HDAC3 and HDAC6 proteins. Our work reveals a novel mechanism of action of those modified compounds and provides a basis for further target-driven modifications of GA as potential cancer therapeutics.

## Results

### Both compound 10d and 10e induce apoptosis in HL-60 cells with downregulation of c-Flip and Mcl-1 and upregulation of Noxa protein

HL-60 cells were treated with varying concentrations of 10d and 10e for 6 h, the percent levels of apoptotic cells was determined using fluorescence-activated cell sorting (FACS) by measuring the percentages of the SubG1 fraction after staining with propidium iodide (PI) (Fig. 1B). More than 70% of the cells in the SubG1 phase were detected after treatment with 1 μM 10d. To reach the same levels of apoptosis induction, 2 μM 10e was required (Fig. 1B). The apoptotic effect of 10e is only slightly less than that of 10d in this cell line, but is in the similar concentration range (1–2 μM). Therefore, decreased ability of compound 10e as compared to 10d to deplete intracellular GSH^2^, does not significantly affect its apoptosis inducing effect.

Western blot analysis was used to measure the apoptosis-related proteins in HL-60 cells treated with 10d and 10e. The cleaved caspase-3, -8, and -9 were detected in HL-60 cells, indicating that both extrinsic and intrinsic apoptotic pathways were involved in apoptosis induction after treatment with both compounds (Fig. 1C). As we reported before^4^, the levels of c-Flip and Mcl-1 were decreased while Bcl-2 was not changed in both 10d and 10e-treated cells (Fig. 1C). The levels of Bak and Bax remained unchanged. BH3-only proteins are a group of proteins that includes, Bim, Noxa, and Puma, known to counteract the anti-apoptotic function of Bcl-2 family. Their regulation has not been tested in CDODO-Me and CDDO-Me treated cells. We measured the levels of Bim, Noxa, and Puma and found that Puma decreased, Noxa increased, while the level of Bim remained unchanged (Fig. 1C). These data suggest that downregulation of c-Flip and Mcl-1, as well as the induction of Noxa is a part of the apoptosis inducing response to treatment with 10d and 10e.

To explore in more detail the effect of 10d and 10e on the three proteins, the cells were treated with 10d or 10e and samples were retrieved at different times and examined for c-Flip and Mcl-1 and Noxa. The timing of apoptosis induction, measured by PARP cleavage, was correlated with a significant decrease in c-Flip and an increase in Noxa proteins (Fig. 1D). We then tested whether the regulation of Mcl-1, c-Flip, and Noxa by 10e was secondary to caspase activation or proteasome degradation. HL-60 cells were pretreated with pan-caspase inhibitor Z-VAD-FMK and q-VD-OPH or proteasome inhibitor MG132. Pretreatment with Z-VAD-FMK or q-VD-OPH followed by 2 μM 10e for 6 h attenuated the reduction in Mcl-1, but not in c-Flip, and did not affect the increase in Noxa level (Fig. 1E). Pretreatment of cells with MG132 enhanced apoptosis of 10e, in spite of the fact that the effects of 10e on c-Flip and Mcl-1 were diminished (Fig. 1F) (MG132 alone induced Noxa and partly induced apoptosis). To prove that MG132 at the used concentration inhibited proteasome, ubiquitinated Mcl-1 was measured using lysates immunoprecipitated with an anti-Mcl-1 antibody and detected in MG132 and MG132 with 10e-treated groups (Fig. 1F). These results suggest that c-Flip is down-regulated through the proteasome-mediated pathway while Mcl-1 level is controlled by both caspase and proteasome-mediated pathways. Importantly, inhibition of proteasome activity appears to induce Noxa.

### Compound 10e is a weaker inhibitor than 10d of AKT and ERK signaling pathways, which are known to regulate Mcl-1 stability

CDDO-Me has been reported to inhibit AKT and mTOR signaling and ERK signaling, both known to regulate Mcl-1 stability through phosphorylation^17^. Components of both signaling pathways were examined after treatment of HL-60 cells with 10d and 10e. The levels of total AKT, p-mTOR, p70S6K, and p-p70S6K, were decreased after treatment with 10d at 1 μM and 10e at 2 μM. Neither 10d nor 10e influenced the levels of ERK and p-ERK. AKT phosphorylates GSK-3β on the Ser^9^ residue that leads to GSK-3β inactivation. Mcl-1 is phosphorylated by GSK-3β at Ser^159^, resulting in its proteasomal degradation^18^. 10d or 10e treatment led to reduction in the levels of phosphorylated GSK-3β on the Ser^9^ without changing GSK-3β protein levels (Sup. Figure 1). These data suggest that compounds 10d and 10e can activate GSK-3β causing Mcl-1 degradation. Compared to 10d, 10e was less effective in reducing Mcl-1 levels. Since Noxa is a protein which selectively binds to and inactivates Mcl-1, apoptosis induction might not depend on the downregulation of Mcl-1 in presence of upregulated Noxa.

### Downregulation of c-Flip by 10e is associated with the inhibition of histone deacetylases and Ku70 acetylation

**Fig. 2:**
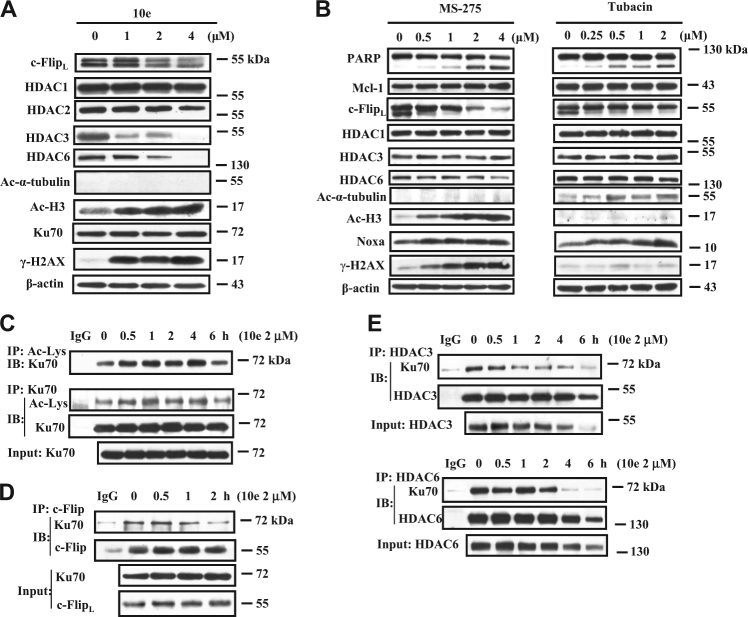
10e decreased protein levels of c-Flip, HDAC3 and 6 and led to Ku70 acetylation. **A** HL-60 cells were treated with the indicated concentrations of 10e for 6 h, then the levels of the indicated proteins were determined using western blot analysis. **B** HL-60 cells were treated with MS-275 or tubacin at the indicated concentrations for 24 h, then relative levels of the indicated proteins were determined by Western blotting. **C**, **D** The whole-cell lysates from HL-60 cells treated with 2 μM 10e for the indicated time were immunoprecipitated with an acetylated lysine antibody, an Ku70 antibody (**C**) or with an c-Flip antibody (**D**), and then immunoblotted with an anti-Ku70, an acetylated lysine or an c-Flip antibody as labeled. **E** The cell lysates from HL-60 cells were immunoprecipitated with an anti-HDAC3 or anti-HDAC6 antibody and then immunoblotted for Ku70, HDAC3, HDAC6 proteins

Inhibition of HDAC has been reported to decrease the levels of c-Flip^19–21^. c-Flip forms a stable complex with Ku70 that is dissociated after Ku70 acetylation^22^. We tested the inhibitory effects of 10d and 10e on the enzymatic activity of purified HDAC and found that, compared to MS-275, a selective inhibitor of HDAC activity, neither compound was inhibitory (Sup. Figure 2). We then tested whether treatment of HL-60 cells with compound 10e has an effect on HDAC protein level. 10e decreased the levels of HDAC3 and HDAC6 proteins but not the levels of HDAC1 and HDAC2 (Fig. 2A). Only the acetylated H3 level, a substrate of HDAC3, was substantially increased; the substrate of HDAC6, α-tubulin was not acetylated (Fig. 2A), suggesting that it is HDAC3 and not HDAC6 activity inhibition that contributes to the downregulation of c-Flip. To confirm this conclusion, we used the relatively specific HDAC inhibitor MS-275, which inhibits HDAC1, 2, and 3^23^, and tubacin, which is a relative selective inhibitor of HDAC6^24,25^. As expected, MS-275 increased the levels of acetylated H3, but not acetylated α-tubulin, while tubacin increased the levels of acetylated α-tubulin, but not acetylated H3 at lower concentrations (Fig. 2B), indicating that it is possible to selectively inhibit either class I HDAC or HDAC6 at controlled concentrations. MS-275 was found to be more effective than tubacin in decreasing the levels of c-Flip and in apoptosis induction as measured by PARP cleavage. Interestingly we found that MS-275, like 10e, decreased the level of HDAC6 but without an increase in α-tubulin acetylation. These data suggest that inhibition of HDAC1, 2, and 3 may lead to a decrease in the level of HDAC6. Inhibition of HDAC6 with tubacin has been reported to cause Ku70 acetylation and c-Flip degradation in colon cancer cells^22^. We increased the concentrations of tubacin and found that it indeed reduced the levels of c-Flip protein in both HL-60 and THP-1 cells. However the reduced levels of c-Flip associated increased levels of acetylated H3 (Sup. Figure 3), suggesting that inhibition of HDAC1, 2, and 3, like MS-275, may contribute to tubacin-induced c-Flip reduction. The effect of compound 10e treatment on Ku70 acetylation was measured by immunoprecipitation (IP) using an acetylated lysine antibody and probed with an anti-Ku70 antibody and found to be increased as well as IP using an anti-Ku70 antibody and probed with the acetylated lysine antibody (Fig. 2C). This treatment also dissociated the c-Flip/Ku70 complex before reduction in the c-Flip protein level (Fig. 2D). We measured the binding interaction of HDAC3 and HDAC6 with Ku70 in cell lysates treated with 10e. The binding of HDAC3 to Ku70 was decreased within 1 h of treatment with 10e while it took 2 h to dissociate the HDAC6/Ku70 complex (Fig. 2E). It has been described that inhibition of HDAC can cause DNA damage and an increased γ-H2AX^26^. We measured the levels of γ-H2AX in HL-60 cells treated with 10e, and found that, like in the treatment with MS-275, the γ-H2AX was increased by 10e but not by tubacin at lower concentrations (Fig. 2A, B). The DNA damage caused by inhibition of HDAC1, 2, and 3, but not HDAC6 may be caused by induced acetylation of Ku70 in nucleus which might also contribute to apoptosis.

**Fig. 3:**
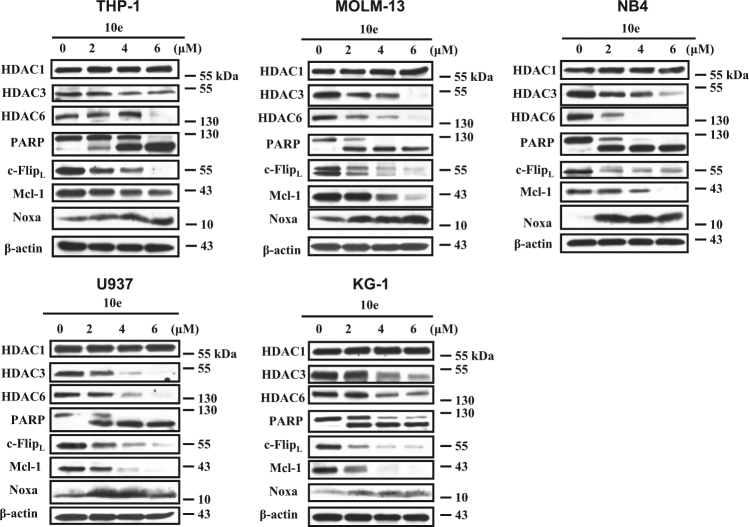
The regulation of 10e on the protein levels of c-Flip, Mcl-1, Noxa, HDAC3, and HDAC6 in different AML cell lines. THP-1, MOLM-13, NB4, U937, and KG-1 cells were treated with 10e at the indicated concentrations for 6 h. The levels of each indicated protein were determined with Western blotting

The findings that 10e treatment of HL-60 cells decreases the levels of c-Flip, Mcl-1, HDAC3, and HDAC6, while increasing the level of Noxa was found to be true in additional AML cell lines, such as THP-1, MOLM-13, NB4, U937, and KG-1 (Fig. 3).

### Noxa plays a crucial role in 10e-induced apoptosis in addition to Mcl-1 and c-Flip downregulation

**Fig. 4:**
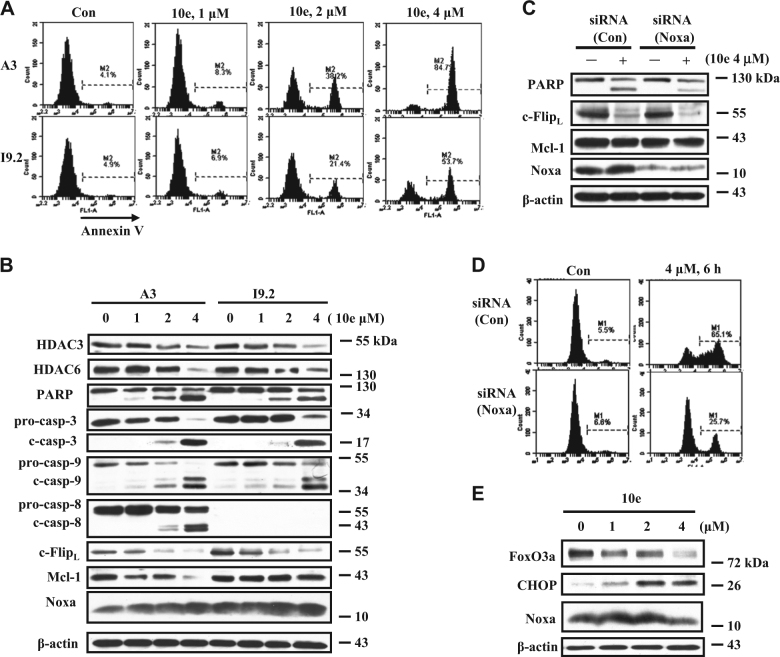
Noxa plays a more important role than c-Flip and Mcl-1 in the apoptosis induction by 10e treatment. **A**, **B** A3 cells expressing caspase-8 and I9.2 cells detecting caspase-8 were treated with 10e at the indicated concentrations for 6 h. Apoptotic cells were determined using FACS analysis after staining with Annexin V (**A**). The relative levels of each protein were measured using specific antibodies with Western blot analysis (**B**). **C**, **D** I9.2 cells were transfected with *Noxa* siRNA for 16 h, then treated with 4 μM 10e for additional 6 h. The levels of PARP, c-Flip_L_, Mcl-1, Noxa, and β-actin were determined by Western blotting (**C**). The apoptotic cells were quantified using FACS after staining with Annexin V-FITC (**D**). **E** I9.2 cells were treated with 10e at the indicated concentration for 6 h, the levels of FoxO3a, CHOP, Noxa, and β-actin were measured by western blot analysis

It has been described that induction of Noxa and downregulation of Mcl-1 causes mitochondria-mediated caspase-9 activation^27^. c**-**Flip is known to block apoptosis through interaction with caspase-8^28^. To compare the roles of caspase-8 and caspase-9 in the apoptosis induced by 10e, Jurkat subclones, A3 (expressing caspase-8) and I9.2 (deleted caspase-8) were used^29^. These cells were treated with 10e at the concentration of 4 μM; in subclone A3 (expressing caspase-8) this treatment caused 84.7% of cells to undergo apoptosis while in the I9.2 cells (deleted caspase-8) 53.7% underwent apoptosis (Fig. 4A) (Apoptosis was measured by FACS analysis based on the Annexin V staining.). Western blot data showed that treatment with 10e decreased the c-Flip protein in both cell lines, while caspase-8 was cleaved in A3 cells. These data suggest that caspase-8 has a certain role in 10e-induced apoptosis since I9.2 cells, with deletion of caspase-8 decreased response to apoptosis induction comparing to A3 cells. The Mcl-1 level was decreased in A3 cells, but not in I9.2 cells after treatment with 10e. The levels of Noxa were similarly induced in both cell lines after 10e treatment. Silencing of Noxa significantly attenuated the apoptosis induced by 10e treatment in I9.2 cells (Fig. 4C), from 65 to 25% (Fig. 4D). Similarly we found that silencing of Noxa attenuated the apoptosis induction by 10e in THP-1 cells (Sup. Figure 4A). These data suggest that Noxa induction but not Mcl-1 downregulation plays another crucial role for apoptosis induction by 10e treatment. Several factors such as FoxO3a and CHOP have been found to regulate the levels of Noxa expression^30^. We found that the levels of FoxO3a were decreased while the levels of CHOP were induced in both I9.2 cells (Fig. 4E) and THP-1 cells (Sup. Figure 4B), suggesting that a CHOP-mediated pathway leads to Noxa induction.

### Both Bak and Bax are activated in 10e-treated cells

**Fig. 5:**
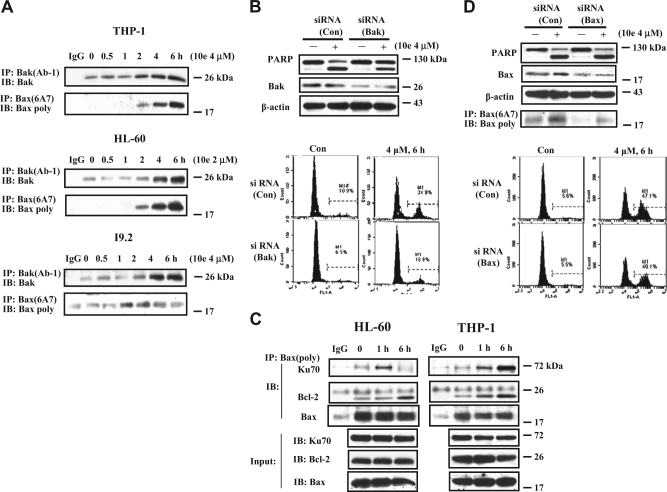
Bak and Bax are activated in 10e-treated cells and contribute to the apoptosis induction. **A** The activated Bak or Bax proteins in THP-1, HL-60, and I9.2 cells treated with 2 μM or 4 μM 10e for the given times were immunoprecipitated with the anti-Bak(Ab-1) or anti-Bax (6A7) antibody (detecting the active forms), respectively, followed by the western blotting using poly anti-Bak or anti-Bax antibody. **B** THP-1 cells were transfected with *Bak* siRNA or a negative *control* siRNA for 30 h, then treated with 4 μM 10e for additional 6 h. The levels of PARP and Bak were determined by western blotting. The apoptotic cells were measured by FACS after staining with Annexin V-FITC. **C** The THP-1 and HL-60 cell lysates treated with 4 μM or 2 μM 10e for 6 h were immunoprecipitated with anti-Bax antibody and immunoblotted with an anti-Ku70, Bax, or Bcl-2 antibody. **D ** THP-1 cells were transfected with *Bax* siRNA or a negative *control* siRNA for 30 h, then treated with 4 μM 10e for additional 6 h. The levels of PARP and Bax were determined by western blotting. The active form of Bax was detected with IP. The apoptotic cells were measured by FACS after staining with Annexin V-FITC

Noxa inactivates Mcl-1 and leads to the activation of Bak. Since silencing of Noxa only partly blocked apoptosis in I9.2 cells treated with 10e (Fig. 4C) and in THP-1 cells (Sup. Figure 4A), it suggested that a non-Noxa/Mcl-1/Bak mediated pathway must also be involved in 10e-induced apoptosis. Using an IP assay, we found that both Bak and Bax were activated in THP-1, HL-60 cells, as well as in I9.2 cells (Fig. 5A). Silencing of Bak, similarly to silencing Noxa (Sup. Figure 4A) attenuated apoptosis induced by 10e in THP-1 cells (Fig. 5B). Bax exists in free and bound forms with Bcl-2 or other proteins^31^. Bax binds to Ku70 and Ku70 acetylation can lead to Bax activation^32,33^. The interaction of Bax with Ku70 or Bcl-2 was assessed by immunoprecipitating Bax and then probing with an anti-Ku70 or anti-Bcl-2 antibody. Interestingly, 10e treatment significantly increased the binding of Bax to Ku70 and Bcl-2 (Fig. 5C). Using the antibody detecting active form Bax, Bax (6A7), these interactions are not found, suggesting the bond form of Bax to Ku70 is not the active form. Silencing of Bax using siRNA decreased Bax protein, but neither the active Bax level nor apoptotic cells decreased in 10e-treated THP-1 cells (Fig. 5D). It has been reported that Ku70 by interacting with Bax stabilizes it by preventing its degradation^33^. We propose that 10e treatment regulates Bax through two ways: (1) increases its binding to non-acetylated Ku70 and (2) leads to Bax activation after dissociation from acetylated Ku70.

## Discussion

Structural modified tritepenoids CDDO-Me and CDODO-Me are potent apoptosis inducers and are being developed for clinical trials of cancer therapy^6,7^. However, their development was delayed due to lack of clearly defined targets. Although, GSH depletion and ROS production have been thought to be the potential mechanisms of apoptosis induction^9–12^, the higher plasma GSH concentration and the role of GSH in redox regulation question GSH as a target^34^. Moreover the high affinity of these compounds to GSH put them into competitive disadvantage. We have found that 10e maintains apoptosis inducing ability of 10d but with decreased binding to GSH^2^. We found that the following mechanisms contribute to the apoptosis induction by 10e: (1) Mcl-1 reduction and Noxa induction; (2) downregulation of c-Flip; and (3) Bax and Bak activation. By further exploring the mechanism causing those changes, we found new targets, HDAC3 and HDAC6. We showed that reduction of HDAC3 and HDAC6 proteins by 10e contribute to the apoptosis induction.

c-Flip plays an important role in regulating extrinsic apoptosis by blocking the activation of caspase-8, while Noxa specifically binds to Mcl-1 and causes apoptosis through mitochondrial-mediated pathway^27,35^. Utilizing Jurkat subclones A3 cells (expressing caspase-8) and I9.2 cells (deleted caspase-8), we showed that 10e decreased the levels of c-Flip in both cell lines, and that I9.2 cells have less response to 10e-induced apoptosis than A3 cells (Fig. 4A), suggesting that c-Flip degradation caused caspase-8 activation play a role in the apoptosis induction. Compared to A3 cells, I9.2 cells contain high level of Mcl-1, which is not decreased by 10e treatment (Fig. 4B). Noxa was induced equally in both A3 and I9.2 cells after 10e treatment (Fig. 4B), but silencing of Noxa significantly decreased apoptosis in 10e-treated I9.2 cells (Fig. 4C) and THP-1 cells (Sup. Figure 4A). These data suggest that Noxa plays a more important role than Mcl-1 and that downregulation of Mcl-1 may not be required once Noxa is induced. This is supported by the observation that MG132 blocks Mcl-1 degradation induced by 10e treatment but enhances apoptosis by inducing Noxa (Fig. 1F).

We found that 10e-induced c-Flip degradation through a new HDAC/Ku70-mediated pathway. c-Flip interacts with Ku70 and is destabilized after Ku70 acetylation, which can be achieved by inhibition of HDAC6^22^. We found that both HDAC3 and HDAC6 bind to Ku70 and dissociate from Ku70 once Ku70 is acetylated following 10e treatment (Fig. 2E). Although, 10e did not inhibit the activity of the pure HDAC enzyme (Sup. Fig. 2), it decreased the protein levels of HDAC3 and HDAC6 (Fig. 2A). Silencing of either HDAC3 or HDAC6 with siRNA augmented 10e-induced apoptosis in THP-1 cells (Sup. Figure 5). Interestingly, acetylated H3, a marker of inhibition of nuclear HDAC including HDAC3, but not the acetylated α-tubulin, a marker of HDAC6 inhibition, was increased (Fig. 2A). These data suggest that inhibiting HDAC3, but not HDAC6, is important in causing Ku70 acetylation and c-Flip degradation. Indeed, we found that the relatively specific inhibitor of HDAC1, 2 and 3, MS-275, decreased the levels of c-Flip, while the HDAC6 inhibitor, tubacin did not cause c-Flip degradation at the concentration only inhibiting HDAC6 based on the acetylated α-tubulin (Fig. 2B). Interestingly MS-275 like 10e treatment decreased the levels of HDAC6 without increasing acetylated α-tubulin (Fig. 2B), suggesting that HDAC3 might regulate the stability of HDAC6, a finding in need of further exploration. Ku70 not only binds to c-Flip but also to Bax. Binding of Ku70 to Bax blocks its activation and translocation to mitochondria^32,36,37^. Ku70 acetylation disassociates Bax and leads to Bax activation^38,39^. We found that treatment with 10e not only increased the levels of activated Bax (Fig. 5A) but also the binding of non-active Bax to Ku70 (Fig. 5C). Since 10e treatment increased the binding of Bax to both Ku70 and Bcl-2 (Fig. 5C), we propose that this is a mechanism protecting a cell from death. It has been found that Ku70 can stabilize Bax through de-ubiquitination^33^. Therefore, 10e treatment simultaneously causes recruitment of Bax to Ku70 and also Bax activation in a portion after Ku70 acetylation. Several HDAC inhibitors have been approved for the treatment of cutaneous T-cell lymphoma^40^. Although, these agents were originally developed as epigenetic regulators, they are potent apoptosis inducers through nonhistone targets. Recently it has been shown that bortezomib, an agent approved for treatment of myeloma, induces apoptosis while decreasing HDAC1, 2 and 3 protein levels^41^. It also has been shown that HDAC3 plays a more important role in cancer cells probably due to localization to the cytoplasm and the nucleus^42^ and its increased levels in some cancer cells^43,44^. Specific inhibitors of HDAC3 are being developed. We found that 10e treatment caused acetylation of Ku70 both in the nucleus and in the cytoplasm (Sup. Figure 6). Acetylation of Ku70 in cytoplasm is the cause of c-Flip degradation and Bax activation while its acetylation in the nucleus is the cause of DNA damage with increased levels of γ-H2AX (Fig. 2A, B).

**Fig. 6:**
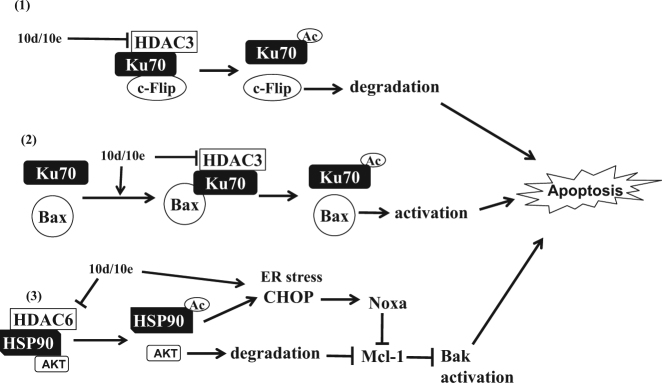
Schematic presentation of apoptosis induction by 10e and 10d. **1** HDAC3/Ku70/c-Flip form a complex which is interrupted by 10e/10d through down-regulating HDAC3, resulting in c-Flip degradation; **2** 10e/10d treatment increases the Ku70/Bax complex formation and, meanwhile, activates Bax by causing Ku70 acetylation through inhibiting HDAC3. **3** 10e/10d treatment causes Noxa induction and AKT degradation by inhibiting or acetylating HSP90 through down-regulating HDAC6. Degraded AKT decreases Mcl-1 protein levels and Noxa induction inactivates Mcl-1, leading to Bak activation. The combined effects of c-Flip downregulation, Bax and Bak activation contribute to the apoptosis induction by 10e/10d treatment

It has been shown that the stability of Mcl-1 is regulated by AKT/GSK-3β signaling and that Noxa is induced by the stress response protein CHOP^45,46^. We found that treatment with 10e induces CHOP (Fig. 4E and sup Fig. 4B) and decreases the AKT level. It has been previously shown that CDDO-Me is a HSP90 inhibitor and inhibition of HSP90 by several agents causes Noxa induction^47,48^. Moreover, AKT is a client protein of HSP90 and the ability of HSP90 binding to client proteins is regulated by HDAC6. Therefore, the downregulation of Mcl-1 and AKT, as well as induction of Noxa may be due to inhibition of HSP90 as a response to decreased HDAC6 levels (Fig. 6). These connections need further study.

In summary, 10e is a novel GA derivative with apoptosis inducing ability and decreased binding to GSH. 10e induces apoptosis through multiple pathways: induction of Noxa and downregulation of c-Flip/Mcl-1, as well as activation of Bax and Bak (Fig. 6). We linked these events to the HDAC/Ku70 axis (Fig. 6). 10e functions as an HDAC3/6 inhibitor by reducing their protein levels rather than inhibiting their enzymatic activity. Our work reveals a novel mechanisms of CDDO-Me or CDODO-Me induced apoptosis which is important for directing further modification and optimization of those compounds as anticancer drugs.

## Materials and methods

### Reagents

10d and 10e were synthesized as we reported before; they were dissolved in dimethylsulfoxide for biological testing^2^. The purity (more than 98%) was determined using HPLC with a Hitachi UV detector L-2400 (Tokyo, Japan). MG132 were purchased from Sigma Chemical Co. (St. Louis, MO, USA). The pan-caspase inhibitor Z-VAD-FMK, MS-275, and tubacin were purchased from Selleckchem (Houston, TX). Antibodies to poly-(ADP-ribose)-polymerase(PARP), pro-caspase-3, and caspase-8 were obtained from BD Biosciences; to β-actin, Bid, Mcl-1, Bcl-2, Bax (6A7), Ku70 (A-9), c-Flip, and Bax were purchased from Santa Cruz Biotechnology, Inc. (Santa Cruz, CA, USA); to procaspas-9, cleaved caspase-9, Bim, Puma, CHOP, FoxO3a, ERK, p-ERK, eIF4E, p-eIF4E, AKT, p-AKT, mTOR, p-mTOR, 4E-BP1, p-4E-BP1, p70S6K, p-p70S6K(Thr^389^), GSK-3β, p-GSK-3β(Ser^9^), Acetyl-α-Tubulin, Bax, Bak, acetylated lysine, and γ-H2AX were purchased from Cell Signaling Technology, Inc. (Berverly, MA, USA); to Noxa and Ku70 were obtained from Abcam, Inc. (Cambridge, MA, USA); to acetyl-Histone H3 was from Active Motif. (Carlsbad, CA, USA), to Bak (Ab-1) was obtained from Merck Millipore (Merck, Darmstadt, Germany). Noxa, Bak, Bax, HDAC3, HDAC6 siRNA, and a control siRNA were purchased from Santa Cruz Biotechnology, Inc.

### Cell culture

Acute myeloid leukemia HL-60, MOLM-13, THP-1, U937, KG-1 cells, and acute promyelocytic leukemia NB4 cells were cultured in RPMI 1640 medium supplemented with 100 units/mL penicillin, 100 μg/mL streptomycin, 1 mmol/L l-glutamine, and 10% (v/v) heat-inactivated fetal bovine serum (FBS). A3 and its caspase-8 deficient I9.2 cells were subclones of human Jurkat cells which were cultured in RPMI 1640 modified to contain 2 mmol/L l-glutamine, 10 mmol/L HEPES, 1.0 mmol/L sodium pyruvate, 4.5 g/L glucose, and 1.5 g/L sodium bicarbonate, 100 units/mL penicillin, 100 μg/mL streptomycin, and 10% (v/v) heat-inactivated FBS.

### Quantitation of apoptotic cells

Apoptotic cells were quantified by FACS analysis after staining with PI and annexin V. For FACS analysis with PI staining, cells were fixed with 70% ice-cold ethanol at a density of 1 × 10^5^ cells/mL and treated by the PI assay kit according to the manufacturer’s instructions (BD Biosciences). For FACS analysis with annexin V staining, Annexin V-FITC Apoptosis Detection Kit (BD Biosciences) was used.

### IP

Cells were collected, washed with PBS and lysed using ice-cold NP-40 lysis buffer (50 mmol/L Tris-HCl (pH 7.5), 150 mmol/L NaCl, 5 mmol/L EDTA, 0.5% NP-40, 50 mmol/L NaF, 0.2 mmol/L Na_3_VO_4_, 1 mmol/L DTT) for 60 min on ice. Total protein (400 µg) was first precleared with 20 µl protein A/G plus-agarose (Santa Cruz Biotechnology) and then subjected to IP with 1–2 µg of either normal IgG or specific antibody at 4 °C for 2 h. Then 20 µl of protein A/G plus-agarose beads were added and incubated overnight to pull down protein-antibody complexes. The beads were spun, washed four times with NP-40 lysis buffer, resuspended in 2X SDS sample buffer (50 mM Tris-HCl (pH 6.8), 2% SDS, 10% glycerol, 5% β-mercaptoethanol) and heated at 98 °C for 5 min for analysis by SDS-polyacrylamide gel electrophoresis and Western blotting.

### Nuclear and cytoplasmic extraction assay

Cytoplasmic and nuclear lysates of HL-60 cells were prepared using NE-PER^®^ Nuclear and cytoplasmic extraction reagent (#78833, Thermo Scientific, Rockford, IL) and subjected to western blot analysis and IP.

Western blot analysis, detection of activated Bak and Bax based conformational changes, as well as siRNA use to silence gene expression were done as we reported before^49^. The HDAC activity was measured as reported before^50^.

## Electronic supplementary material

Supplementary Figure Legends

Supplementary Figures
