# Supplementary material for: HDAC and Ku70 axis- an effective target for apoptosis induction by a new 2-cyano-3-oxo-1,9-dien glycyrrhetinic acid analogue
Source: Cell Death Dis. 2018 May 24;9(6):623. doi: 10.1038/s41419-018-0602-1 (PMC5967349; doi:10.1038/s41419-018-0602-1)
Supplement: Supplementary file 1 — Supplementary Figure Legends [file 41419_2018_602_MOESM1_ESM.docx]

**Supplemental Figure 1. 10d and 10e inhibit AKT signaling which contributes to the reduction of Mcl-1 protein in HL-60 cells.** HL-60 cells were treated with 10d or 10e for 6 h and the levels of the indicated proteins were determined with Western blot analyses using specific antibodies.

**Supplemental Figure 2. 10d and 10e do not inhibit the enzymatic activity of HDACs.** Using HeLa nuclear extracts as HDAC enzyme resource, the HDAC inhibition ability was measured as reported in ref 50.

**Supplemental Figure 3. Tubacin at higher concentrations reduced the levels of c-Flip associating with increased levels of actylated α-tubulin and acetylated H3 in both HL-60 and THP-1 cells.** HL-60 and THP-1 cells were treated with 10e at the indicated concentrations for 24 h. The levels of each indicated protein were determined with Western blotting analysis.

**Supplemental Figure 4. 10e-induced Noxa associates with CHOP induction and that silencing of Noxa attenuates 10e-induced apoptosis in THP-1 cells.** **A,** THP-1 cells were transfected with *Noxa* siRNA or a negative *control* siRNA for 16 h, following treatment with 4 μM 10e for additional 6 h. The levels of PARP, c-Flip_L_, Mcl-1, Noxa and β-actin were determined with Western blotting. **B,** THP-1 cells were treated with 10e at the indicated concentrations for 6 h, the levels of FoxO3a, CHOP, Noxa and β-actin were measured with Western blot analysis.

**Supplemental Figure 5. Silencing of HDAC3 and HDAC6 enhanced 10e-induced apoptosis in THP-1 cells.** THP-1 cells were transfected with *HDAC3*, *HDAC6* siRNA or a negative *control* siRNA for 16 h, then treated with 4 μM 10e for additional 4 h. The levels of HDAC3, HDAC6, PARP, c-Flip_L_, Noxa, Ac-H3, Ac-tubulin and β-actin were determined by Western blotting.

**Supplemental Figure 6. 10e causes Ku70 acetylation in both nucleus and cytoplasm in HL-60 cells.** Nuclear and cytoplasmic extracts from HL-60 cells treated with or without 2 μM 10e for 2 h were immunoprecipitated with an anti-acetylated lysine antibody and probed with Ku70 antibody. HDAC2 and GADPH were used to indicate the purity of nucleus and cytoplasm.
