# Supplementary material for: HDAC and Ku70 axis- an effective target for apoptosis induction by a new 2-cyano-3-oxo-1,9-dien glycyrrhetinic acid analogue
Source: Cell Death Dis. 2018 May 24;9(6):623. doi: 10.1038/s41419-018-0602-1 (PMC5967349; doi:10.1038/s41419-018-0602-1)
Supplement: Supplementary file 2 — Supplementary Figures [file 41419_2018_602_MOESM2_ESM.pptx]

## Slide 1
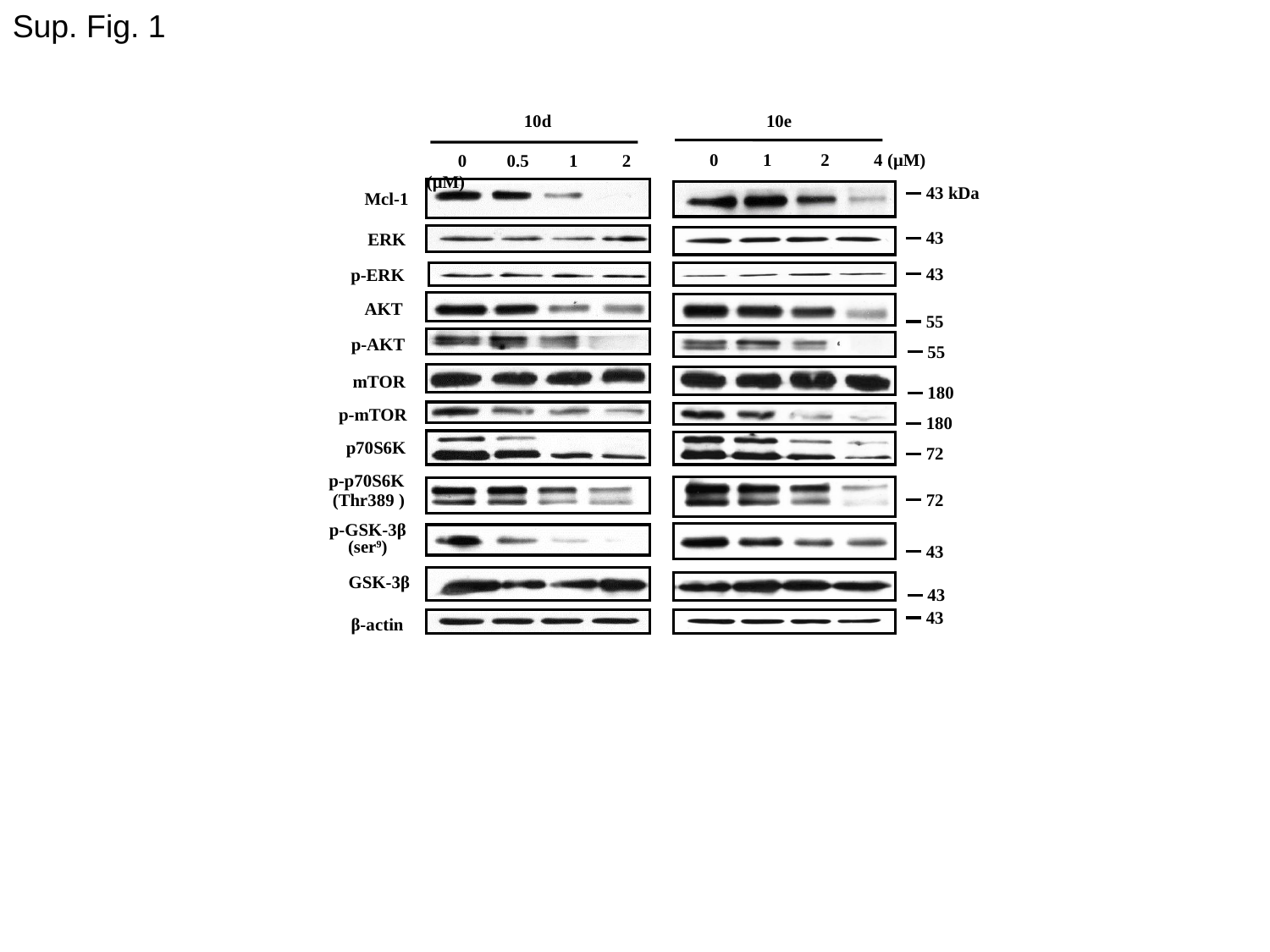

Sup. Fig. 1
10d
 0 0.5 1 2 (μM)
10e
 0 1 2 4 (μM)
Mcl-1
ERK
p-ERK
AKT
p-AKT
mTOR
p-mTOR
p70S6K
p-p70S6K
(Thr389 )
p-GSK-3β
(ser9)
GSK-3β
β-actin
43 kDa
43
43
55
55
180
180
72
72
43
43
43

## Slide 2
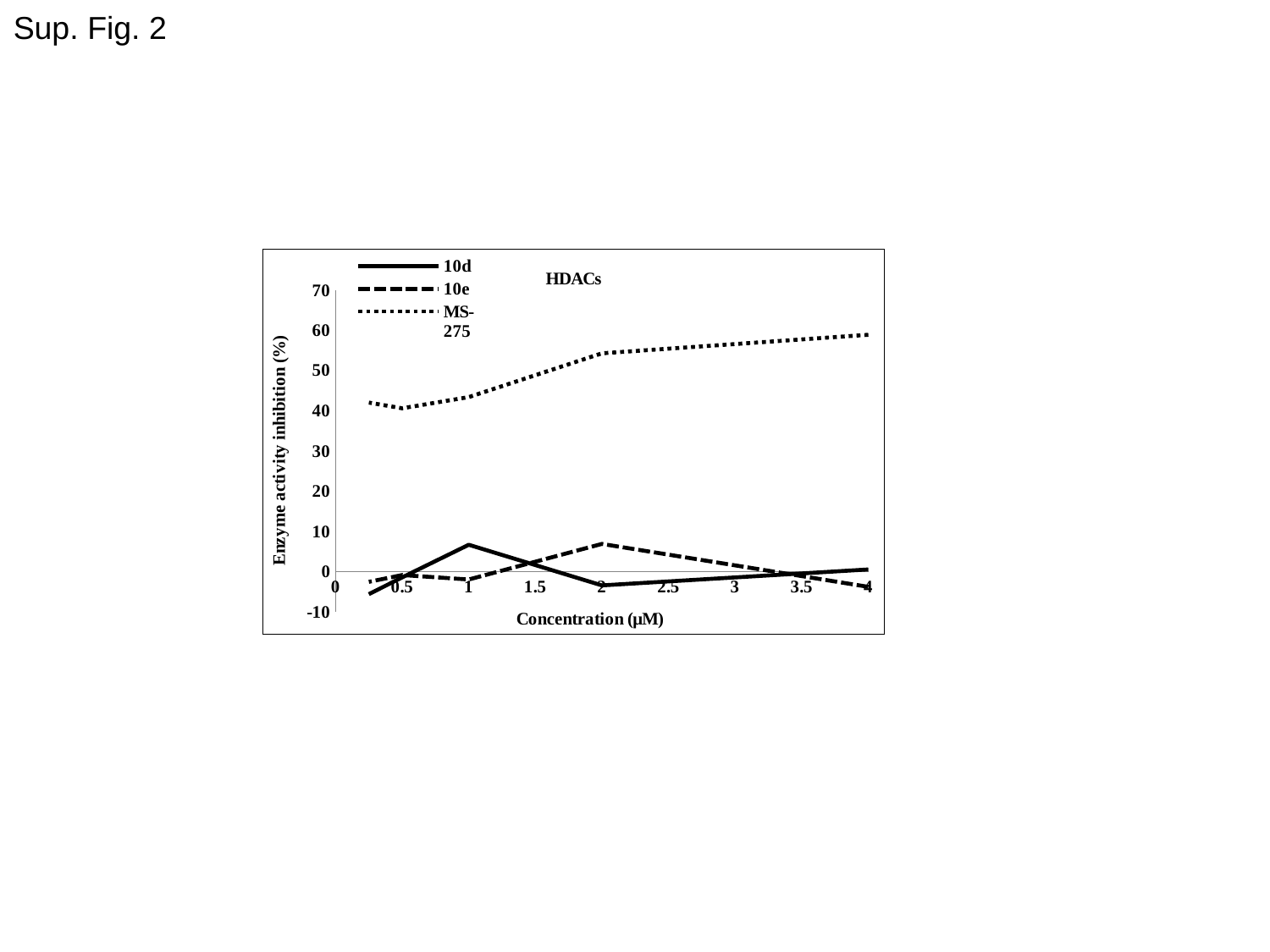

Sup. Fig. 2
### Chart: HDACs
| Category | 10d | 10e | MS-275 |
|---|---|---|---|

## Slide 3
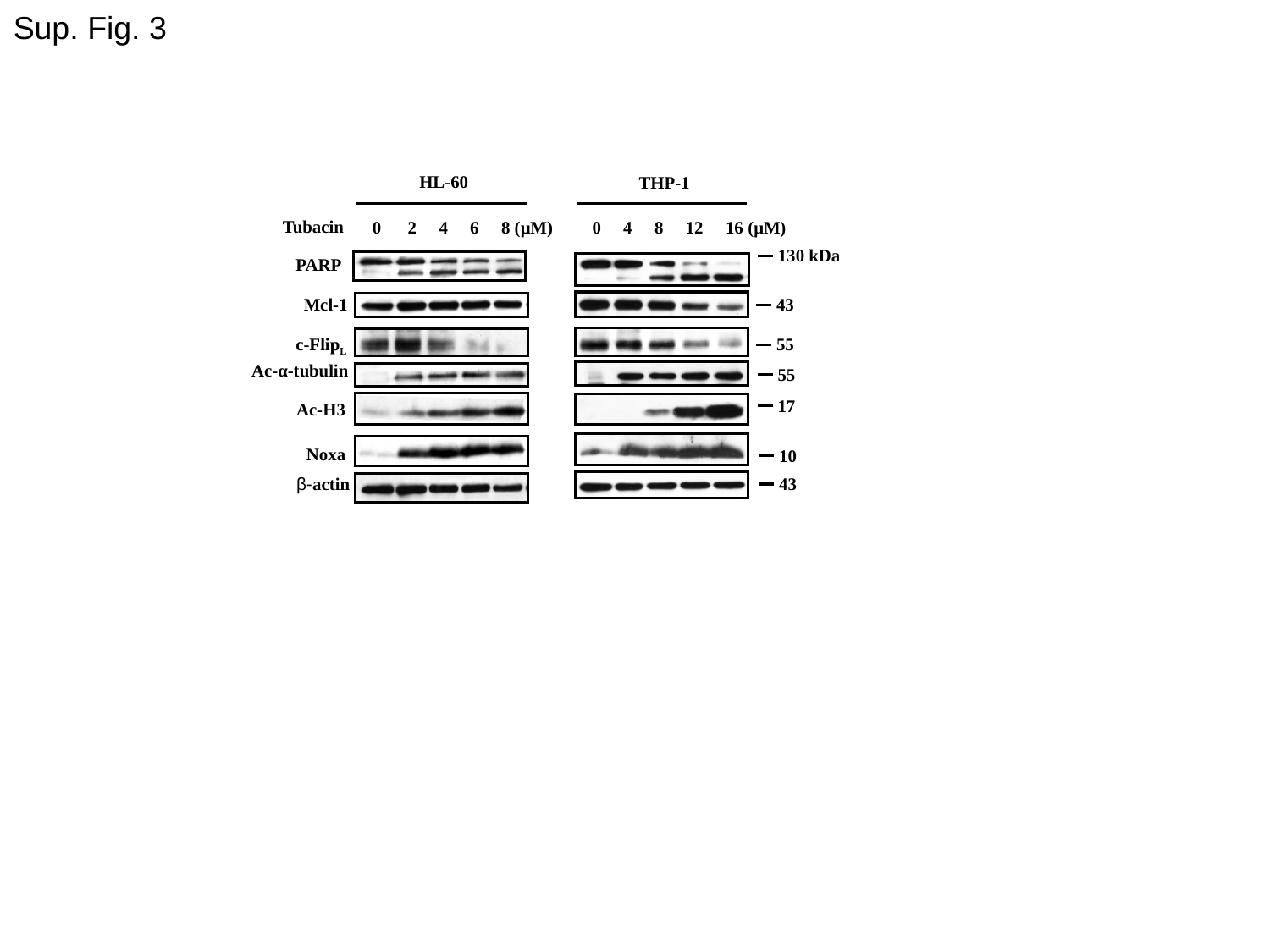

Sup. Fig. 3
HL-60
THP-1
 0 2 4 6 8 (μM)
 0 4 8 12 16 (μM)
130 kDa
PARP
Mcl-1
43
c-FlipL
55
Ac-α-tubulin
55
17
Ac-H3
Noxa
10
43
β-actin
Tubacin

## Slide 4
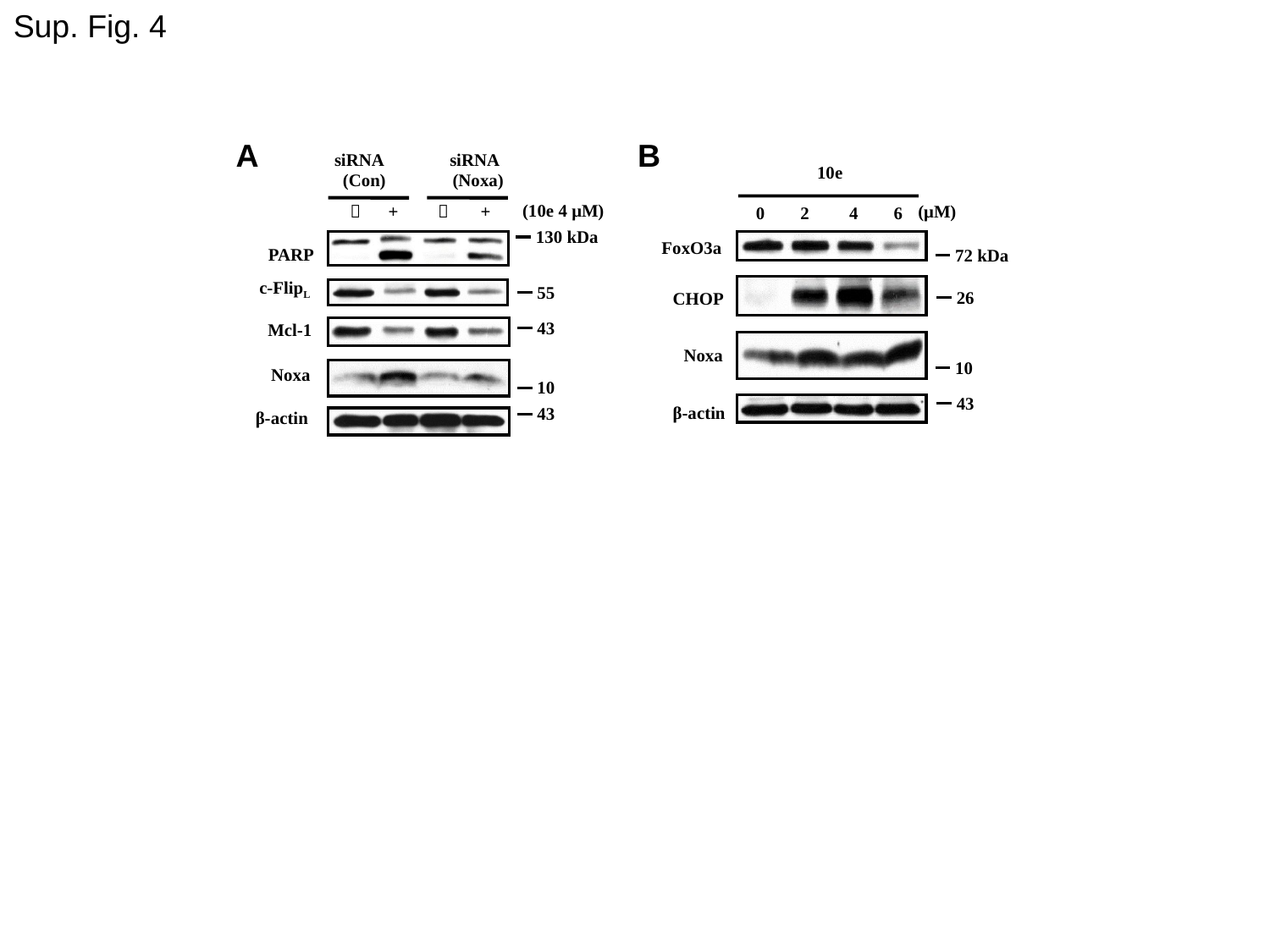

Sup. Fig. 4
A
B
10e
 0 2 4 6
FoxO3a
CHOP
Noxa
β-actin
72 kDa
26
10
43
 (μM)
siRNA siRNA
 (Con) (Noxa)
 － + － +
PARP
Mcl-1
Noxa
β-actin
c-FlipL
130 kDa
55
43
10
43
(10e 4 μM)

## Slide 5
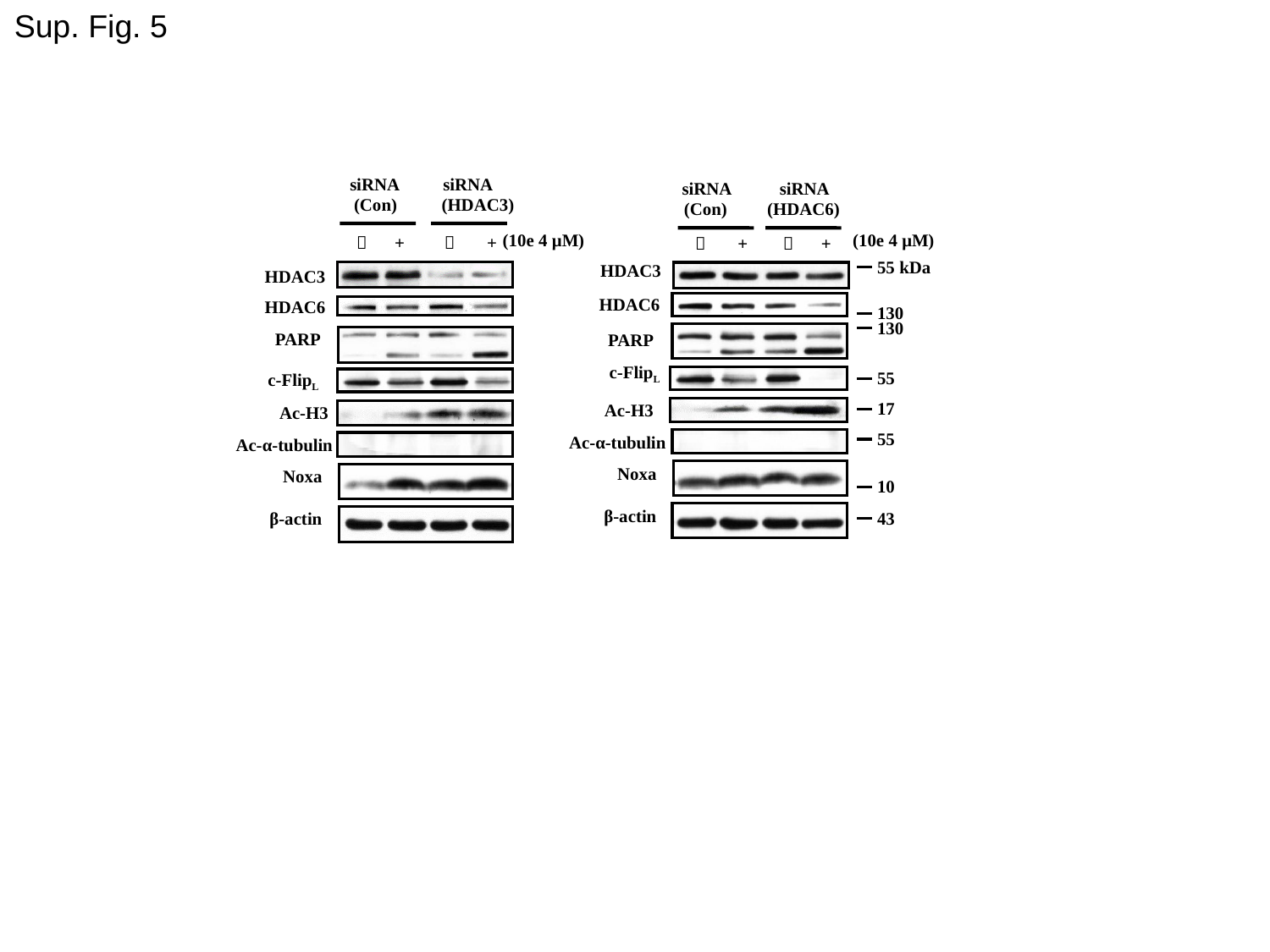

Sup. Fig. 5
siRNA siRNA
 (Con) (HDAC3)
 － + － +
HDAC3
HDAC6
PARP
Ac-H3
Ac-α-tubulin
Noxa
β-actin
c-FlipL
c-FlipL
(10e 4 μM)
siRNA siRNA
 (Con) (HDAC6)
 － + － +
HDAC6
PARP
Ac-H3
Ac-α-tubulin
Noxa
β-actin
HDAC3
(10e 4 μM)
55 kDa
130
130
55
17
55
10
43

## Slide 6
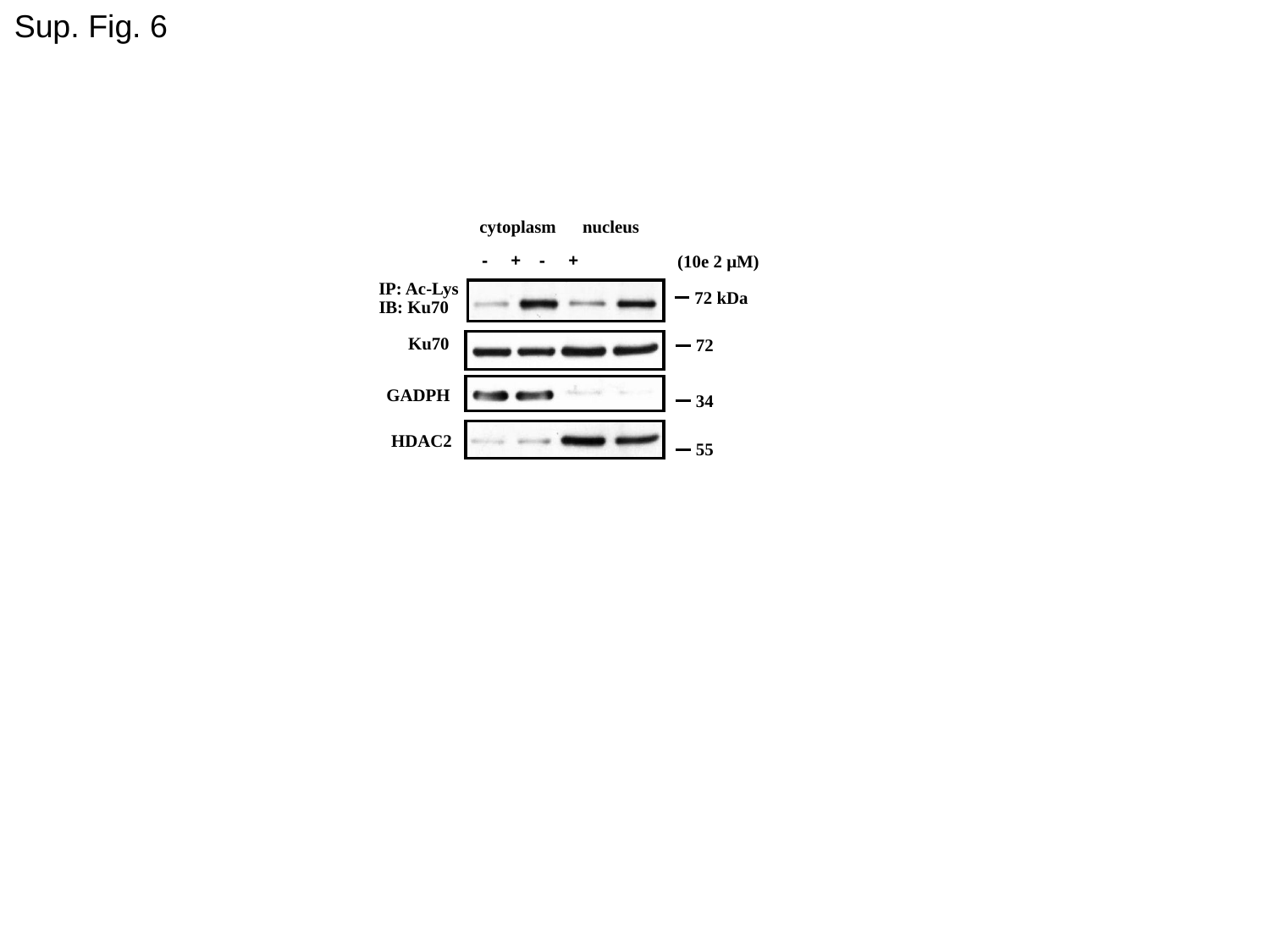

Sup. Fig. 6
 cytoplasm nucleus
 - + - +
(10e 2 μM)
 IB: Ku70
Ku70
GADPH
HDAC2
IP: Ac-Lys
72 kDa
72
34
55
